# Disparities in Children’s Blood Lead and Mercury Levels According to Community and Individual Socioeconomic Positions

**DOI:** 10.3390/ijerph120606232

**Published:** 2015-05-29

**Authors:** Sinye Lim, Mina Ha, Seung-Sik Hwang, Mia Son, Ho-Jang Kwon

**Affiliations:** 1 Department of Occupational and Environmental Medicine, Kyung Hee University Hospital, 23 Kyungheedae-Ro, Dongdaemun-Gu, Seoul 130-872, Korea; E-Mail: drforest@hanmail.net; 2 Department of Preventive Medicine, Dankook University College of Medicine, 119 Dandae-Ro, Dongnam-Gu, Cheonan, Chungnam 330-714, Korea; E-Mail: hojang@dku.edu; 3 Department of Social Medicine, Inha University School of Medicine, 366 Seohae-Daero, Jung-Gu, Incheon 400-712, Korea; E-Mail: cyberdoc@inha.ac.kr; 4 Department of Preventive Medicine, Kangwon National University School of Medicine, 1, Gangwondaehak-Gil, Chuncheon, Gangwon 200-701, Korea; E-Mail: sonmia@empal.com

**Keywords:** socioeconomic position, children, blood lead, blood mercury, community intervention

## Abstract

We aimed to examine the associations between blood lead and mercury levels and individual and community level socioeconomic positions (SEPs) in school-aged children. A longitudinal cohort study was performed in 33 elementary schools in 10 cities in Korea. Among a total of 6094 children included at baseline, the final study population, 2281 children followed-up biennially, were analyzed. The geometric mean (GM) levels of blood lead were 1.73 μg/dL (range 0.02–9.26) and 1.56 μg/dL (range 0.02–6.83) for male and female children, respectively. The blood lead levels were significantly higher in males, children living in rural areas, and those with lower individual SEP. The GM levels of blood mercury were 2.07 μg/L (range 0.09–12.67) and 2.06 μg/L (range 0.03–11.74) for males and females, respectively. Increased blood mercury levels were significantly associated with urban areas, higher individual SEP, and more deprived communities. The risk of high blood lead level was significantly higher for the lower individual SEP (odds ratio (OR) 2.18, 95% confidence interval (CI) 1.36–3.50 in the lowest educational attainment of the father), with a significant dose-response relationship observed after adjusting for the community SEP. The association between high blood lead levels and lower individual SEP was much stronger in the more deprived communities (OR 2.88, 95% CI 1.27–6.53) than in the less deprived communities (OR 1.40, 95% CI 0.76–2.59), and showed a significant decreasing trend during the follow-up only in the less deprived communities. The risk of high blood mercury levels was higher in higher individual SEP (OR 0.64, 95% CI 0.40–1.03 in the lowest educational attainment of the father), with a significant dose-response relationship noted. Significant decreasing trends were observed during the follow-up both in the less and more deprived communities. From a public health point-of-view, community level intervention with different approaches for different metals is warranted to protect children from environmental exposure.

## 1. Introduction

Children are more vulnerable to adverse health outcomes from environmental exposure than adults. From the prenatal period to adolescence, children are exposed to different environmental exposure sources via their diets and behaviors such as hand-to-mouth behaviors and indoor activities [1]. In addition, children drink more water, breathe more air, and consume more food per kg body weight than adults in order to meet their higher metabolic rates [2]. Furthermore, children grow rapidly and have immature detoxification systems, which make them particularly susceptible to various toxicants.

Socioeconomic position (SEP) is an important factor in children’s health, with a low SEP linked to increased exposure and absorption of environmental contaminants, and enhanced toxic effects [3]. Children who live in socioeconomically disadvantaged environments could be exposed to various hazards through substandard housing conditions, houses with lead-based paint, persistent organic pollutants, air pollution, noise, mold, pesticides, and secondhand smoke [3,4,5,6,7]. Further, their houses are more likely to be located in environmentally polluted places, such as near highways, intersections, municipal waste sites or incinerators, and industrial facilities [1]. Low SEP communities play an important role in increasing the burden of exposure to environmental contaminants through direct and indirect mechanisms with the physical, chemical, psychological, and social environments where children are living. These communities are characterized by a lack of green spaces, lower accessibility to health care services, more psychologically stressful environments, high crime rates, poorer health-related behaviors, lack of social networks, and higher levels of problematic child behaviors [1,8].

Among several environmental contaminants, lead and mercury are known to play important roles in children’s environmental health. Most developed countries have set up national policies to reduce the body burden of these toxicants, and as a result, these levels have been declining. However, low-level exposure, as well as intoxication, has substantial impacts on children’s health. For example, intellectual impairments have been reported for exposure below 10 μg/dL of blood lead [9].

Children are exposed to lead mainly by tranfer from their hands, which are contaminated by lead-containing dust and soil, to their mouths. The proportion of ingested lead, which is absorbed through the gastrointestinal system, among the total body burden is higher in children (40%–50%) than in adults (3%–10%). On the other hand, the highest percentage of body burden is stored in the bone and teeth, which is less in children than adults [10]. The half-life of lead is up to 27 years in the bones and teeth, and approximately 30 days in the blood; the blood lead level has been reported to show a decreasing trend as children get older, largely owing to extension of the body volume during growth [11].

Mercury, a well-known global pollutant, exists in three forms: elemental, inorganic, and organic. Methylmercury, an organic form of mercury is important in terms of health outcomes. Several biological specimens have been used for measuring the mercury body burden in the general population, including blood and hair for organic mercury and urine for inorganic mercury [12]. In large-scale epidemiologic studies, total mercury has been used as a proxy of organic mercury. The half-lives of mercury in the blood are 58 days for elemental mercury, 70–80 days for methylmercury, and 1–2 months for inorganic mercury. Common exposure sources of methylmercury are breast milk for newborns and infants, and fish consumption for the general population [13], and even low levels of mercury can cause adverse health effects [12,14]. In Korea, a previous study reported that the mercury body burden is especially high in people consuming raw fish frequently and with high monthly household income [14]. However, there was not sufficient evidence for determining an association with SEP.

In Korea, although some studies have examined health equity in children [15,16], studies to examine environmental exposure disparities have been rarely performed. This study aimed to determine if there was an association between blood lead and mercury levels and socioeconomic factors in Korean children at the community and individual levels, and if the patterns of the levels according to children’s growth differed according to different individual and community level SEPs, using longitudinal follow-up data.

## 2. Materials and Methods

### 2.1. Study Population

The Children’s Health and Environment Research (CHEER) study aims to explore the association between exposure to environmental pollutants and its health effects. It was designed as a cohort study, and the study population was enrolled mostly in 2005–2006, namely the first wave (baseline). The enrolled children in the first wave were 1st or 2nd grade elementary school students and were followed up biennially along with another two waves in 2007–2008 and 2009–2010 (Figure S1). The study design and preliminary results of the CHEER study have been described elsewhere [17]. Among the baseline population of 6094 children who had undergone measurements of the blood metal values, 2313 children (38.0%) were followed-up completely throughout the three waves. After excluding 32 children (28 without individual SEP information, two nine-year-olds at enrollment, and two outliers of blood metal levels), we constructed the final study population with 2281 children. Among children who were in the 1st or 2nd grade in elementary schools in 2005–2006 and who were originally targeted to follow-up, the children examined at all waves completely, compared to those lost to follow-up (final follow-up rate: 52.2%), were more likely to be female, living in urban areas, and have lower household income (Table S1).

Thirty-three elementary nationwide schools in 10 cities located in urban (seven schools in three cities), industrial complex (hereafter “industrial”, 12 schools in four cities), and rural (14 schools in three cities) areas in Korea participated. Because of the small size of the schools in rural areas, more schools were included. An urban area was classified as a city in a metropolitan area without industrial complexes in the vicinity. An industrial area was defined as an area with an industrial complex nearby, regardless of the population size. Rural areas were defined as agricultural regions with relatively small populations. The participating schools were not randomly selected because of study feasibility and because the primary purpose of the study was not to produce reference values of blood metal levels, but rather to explore the associations with health effects.

The study protocol was approved by the Institutional Review Board of Dankook University Hospital and informed consent was obtained from the parents or guardians of the participating children prior to enrollment.

### 2.2. Socioeconomic Position Indicators

To gather information on the individual SEP indicators, a questionnaire was administered to the parents or guardians of each child. As the individual SEP indicators, both the household total monthly income in KRW (<2000; 2000 < 3000; ≥3000 (×10^3^) KRW; 1 USD = 1132 KRW as of 4 January 2010) and the father’s educational attainment (less than high school; graduated from high school; entrance to or graduation from college or university) were used, which have been validated in previous studies [15,16].

As the community SEP indicator, we used the deprivation index (DI) [18], which was developed by modifying the Carstairs deprivation score [19] and Townsend index [20]. The DI was constructed using five variables: household overcrowdedness, male unemployment rate, head of the family in lower socioeconomic status, home ownership, and substandard living resources. Each score was summed and z standardized, categorized evenly into five groups, and subsequently allocated a community-specific DI. A higher DI indicates increasing deprivation. The DI was calculated from all administrative districts nationwide, using data from the 2005 National Census [21]. The area DI was stratified into two levels: more deprived communities (DI > 1.00) and less deprived communities (DI ≤ 1.00).

### 2.3. Blood Lead and Mercury Measurements

Five mL of venous blood were withdrawn in a heparin-coated tube. Whole blood specimens were stored at −20 °C until analysis. The blood lead levels were measured using a SpectrAA-800 atomic absorption spectrophotometer equipped with a graphite furnace and Zeeman correction (Varian, Mulgrave, VIC, Australia) at a laboratory. The total blood mercury level was determined in the same laboratory by cold vapor atomic absorption spectrophotometry (M-6000A apparatus; CETAC, Omaha, NE, USA).

The laboratory has undertaken extensive internal and external quality assurance programs. The limit of detection (LOD) for lead and mercury were 0.03 μg/dL and 0.01 μg/L, respectively. When the lead and mercury levels were below the LOD, the values were replaced by 1/√2 × LOD [22].

### 2.4. Confounders and Covariates

Potential confounders and covariates associated with the body burden of heavy metal and exposure factors were selected based on the results of the univariate analyses in the study population and reviews of the literature. The information for confounding factors and covariates was obtained through a questionnaire and included age [23], gender [24], secondhand smoke (yes, no) [25,26], residential area (urban, industrial, rural), hours playing outside (<1 h/day, ≥1 h/day for lead) [10], and frequency of fish consumption per week (≤1 time/week, 2–3 times/week, ≥4 times/week for mercury) [13]. The children’s weight (kg) and height (cm) were measured at each survey.

### 2.5. Statistical Analyses

Logistic regression analysis was used to estimate the risks of elevated lead and mercury levels, which were defined as the median blood level or higher, according to the levels of individual SEP, after adjusting for age, gender, and secondhand smoke. The community SEP (DI) was adjusted for or stratified by in the models. To determine the dose-response relationships between the lead and mercury levels and individual SEP level, we calculated the *p*-values using the ordinal scale of the SEP indicator in the corresponding model. To determine if there was an interaction effect of community SEP on the associations between the lead and mercury levels and individual SEP, we obtained the *p*-values of the interaction terms of individual and community SEP variables in the corresponding full model. We applied a linear mixed model adjusted for the covariates to test the differences of patterns of lead and mercury levels during the follow-up (as the children grew older) by different individual SEPs, which were stratified by community SEP. All analyses were conducted using SAS 9.1 software (SAS Institute, Cary, NC, USA), with a *p*-value of <0.05 indicating statistical significance.

## 3. Results

The geometric mean (GM) levels of blood lead were 1.73 μg/dL (range 0.02~9.26) for male and 1.56 μg/dL (range 0.02~6.83) for female children (Table 1). The 5th and 95th percentiles of blood lead were 0.72 and 3.55 μg/dL in male, and 0.62 and 3.31 μg/dL in female children, respectively. The blood lead levels were significantly higher in males, during the first survey year, in children living in rural areas, and in children with lower household total monthly income (household income, hereafter) and lower father’s educational attainment. The blood lead level was significantly higher in children who spent more time playing outdoors (GM: 1.54, 1.65, and 1.93 μg/dL for children playing outside <1, 1 < 5, and ≥5 hs a day, respectively).

The GM levels of blood mercury were 2.07 (range 0.09–12.67) for male and 2.06 μg/L (range 0.03–11.74) for female children (Table 2). The 5th and 95th percentiles of blood mercury were 0.53 and 5.21 μg/L in male, and 0.68 and 5.25 μg/L in female children, respectively. 

**Table 1:** Blood lead distributions at enrollment according to basic characteristics and socioeconomic positions of 2281 children, CHEER, 2005–2006, Korea.

Variable		Male								Female							
		Number of Children	GM * (ug/dL)	Range	Percentiles					Number of Children	GM * (ug/dL)	Range	Percentiles				
				(ug/dL)	5th	25th	50th	75th	95th			(ug/dL)	5th	25th	50th	75th	95th
Total		1136	1.73 ^†^	0.02–9.26	0.72	1.33	1.85	2.48	3.55	1145	1.56	0.02–6.83	0.62	1.22	1.65	2.25	3.31
Enrolled year	2005	447	1.83 ^‡^	0.29–7.67	0.71	1.37	1.97	2.58	3.56	423	1.78 ^†^	0.10–6.83	0.71	1.38	1.92	2.48	3.55
	2006	689	1.68	0.02–9.26	0.74	1.31	1.75	2.39	3.51	722	1.45	0.02–6.51	0.57	1.15	1.54	2.07	3.07
Age at enrollment (years)	6	213	1.79 ^‡^	0.04–7.67	0.77	1.33	1.96	2.46	3.51	238	1.62 ^‡^	0.24–6.83	0.63	1.25	1.68	2.27	3.37
	7	733	1.68	0.02–9.26	0.67	1.29	1.79	2.40	3.51	718	1.51	0.02–6.51	0.58	1.19	1.60	2.22	3.24
	8	190	1.90	0.45–6.29	0.81	1.51	1.94	2.65	3.63	189	1.71	0.10–4.97	0.81	1.35	1.79	2.34	3.55
Residential area	Urban	385	1.70 ^‡^	0.27–6.29	0.75	1.31	1.78	2.37	3.25	372	1.50 ^†^	0.10–6.51	0.62	1.17	1.55	2.08	3.15
	Industrial	433	1.66	0.02–9.26	0.58	1.25	1.79	2.48	3.61	434	1.47	0.02–4.74	0.49	1.19	1.61	2.28	3.24
	Rural	318	1.90	0.42–7.67	0.83	1.45	2.00	2.58	3.57	339	1.75	0.23–6.83	0.71	1.39	1.82	2.45	3.51
Household income (×103KRW/month) ^‡^	<2000	379	1.91 ^†^	0.02–9.26	0.77	1.45	2.06	2.65	3.83	398	1.67 ^‡^	0.04–6.83	0.67	1.29	1.73	2.33	3.38
	2000–2999	418	1.62	0.04–4.45	0.65	1.27	1.76	2.36	3.37	406	1.56	0.02–4.30	0.62	1.22	1.72	2.29	3.18
	≥3000	339	1.70	0.26–5.29	0.69	1.28	1.75	2.42	3.56	341	1.45	0.05–6.51	0.56	1.13	1.53	2.07	3.07
Educational attainment of father (years)	<12	47	2.30 ^‡^	0.83–9.26	1.01	1.69	2.40	3.06	5.04	57	1.93 ^‡^	0.31–4.98	0.47	1.41	2.27	2.89	4.03
	12	494	1.72	0.02–5.29	0.65	1.33	1.90	2.48	3.50	479	1.58	0.03–6.83	0.63	1.26	1.70	2.27	3.24
	>12	521	1.67	0.04–5.01	0.75	1.28	1.76	2.38	3.38	547	1.49	0.02–6.51	0.61	1.16	1.57	2.15	3.22
Deprivation index ^§^	≤1.00	694	1.76	0.02–9.26	0.66	1.34	1.93	2.57	3.64	698	1.57	0.02–6.83	0.56	1.23	1.72	2.36	3.37
	>1.00	442	1.69	0.42–5.07	0.82	1.27	1.75	2.28	3.19	447	1.55	0.10–4.97	0.70	1.22	1.57	2.07	3.15

* Calculated using the least square means of the log transformed lead and mercury levels, adjusted for age, gender, child’s weight, residential area, second hand smoke, survey year, hours for playing outside (for lead), and frequency of fish consumption per week (for mercury). ^†^ *p* < 0.0001 or ^‡^ *p* < 0.05 calculated by *t*-test or one-way ANOVA (analysis of variance). ^§^ 1 USD equals 1132 KRW as of 4 January 2010. ^ǁ^ Deprivation index of the community was calculated from household overcrowdedness, male unemployment rate, head of family in low socioeconomic position, house ownership, and substandard living resources based on data from the 2005 Census. Numbers do not always have the same total because of the missing value. CHEER, Children’s Health and Environmental Research; GM, geometric mean; SEP, socioeconomic position.

**Table 2:** Blood mercury distributions at enrollment according to basic characteristics and socioeconomic positions of 2281 children, CHEER, 2005–2006, Korea.

Variable		Male								Female							
		Number of Children	GM * (ug/dL)	Range	Percentiles					Number of Children	GM * (ug/dL)	Range	Percentiles				
				(ug/dL)	5th	25th	50th	75th	95th			(ug/dL)	5th	25th	50th	75th	95th
Total		1136	2.07	0.09–12.67	0.53	1.45	2.27	3.47	5.21	1145	2.06	0.03–11.74	0.68	1.42	2.21	3.31	5.25
Enrolled year	2005	447	2.47 ^†^	0.11–11.20	0.70	1.94	2.79	3.88	5.20	423	2.56 ^†^	0.08–11.50	0.84	1.93	2.91	3.87	5.28
	2006	689	1.84	0.09–12.67	0.47	1.28	1.93	3.04	5.21	722	1.82	0.03–11.74	0.61	1.23	1.88	2.81	4.87
Age at enrollment (years)	6	213	2.10	0.13–9.92	0.66	1.48	2.21	3.30	5.23	238	2.01	0.03–9.17	0.71	1.39	2.17	3.25	5.27
	7	733	2.02	0.09–12.67	0.53	1.41	2.19	3.42	5.21	718	2.01	0.03–11.74	0.66	1.37	2.13	3.15	5.27
	8	190	2.23	0.12–7.32	0.43	1.57	2.64	3.74	5.10	189	2.36	0.08–11.50	0.71	1.78	2.78	3.67	5.25
Residential area	Urban	385	2.56 ^†^	0.12–12.67	0.80	1.93	2.82	3.91	5.86	372	2.56 ^†^	0.09–11.74	0.88	1.77	2.78	3.87	5.99
	Industrial	433	1.87	0.09–10.93	0.43	1.28	2.04	3.21	5.09	434	1.76	0.03–10.53	0.55	1.20	1.84	2.93	4.87
	Rural	318	1.83	0.11–6.74	0.49	1.29	1.93	2.98	4.72	339	1.99	0.08–6.88	0.72	1.45	2.19	3.15	4.69
Household income (×103KRW/month) ^‡^	<2000	379	2.11	0.09–12.67	0.59	1.49	2.30	3.44	5.23	398	1.97 ^‡^	0.03–11.74	0.59	1.40	2.20	3.21	4.61
	2000–2999	418	1.99	0.15–11.20	0.46	1.35	2.18	3.42	5.17	406	2.08	0.07–9.17	0.76	1.44	2.19	3.30	5.49
	≥3000	339	2.11	0.12–10.91	0.56	1.48	2.32	3.62	5.25	341	2.15	0.10–11.50	0.70	1.45	2.29	3.53	5.74
Educational attainment of father (years)	<12	47	1.76 ^‡^	0.26–7.97	0.44	1.01	1.97	2.74	5.19	57	1.97	0.31–8.34	0.50	1.41	2.14	3.09	4.46
	12	494	1.96	0.09–9.98	0.46	1.39	2.19	3.30	5.00	479	1.94	0.03–10.53	0.61	1.31	2.13	3.30	4.89
	>12	521	2.27	0.12–12.67	0.70	1.64	2.47	3.76	5.53	547	2.21	0.03–11.50	0.82	1.54	2.32	3.46	5.41
Deprivation index ^§^	≤1.00	694	2.06	0.09–10.93	0.57	1.45	2.22	3.46	5.17	698	1.95 ^‡^	0.18–11.74	0.85	1.55	2.32	3.53	5.49
	>1.00	442	2.08	0.11–12.67	0.46	1.45	2.31	3.47	5.55	447	2.25	0.03–10.53	0.59	1.34	2.14	3.18	4.99

* Calculated using the least square means of the log transformed lead and mercury levels, adjusted for age, gender, child’s weight, residential area, second hand smoke, survey year, hours for playing outside (for lead), and frequency of fish consumption per week (for mercury). ^†^ *p* < 0.0001 or ^‡^ *p* < 0.05 calculated by *t*-test or one-way ANOVA (analysis of variance). ^§^ 1 USD equals 1132 KRW as of 4 January 2010. ^ǁ^ Deprivation index of the community was calculated from household overcrowdedness, male unemployment rate, head of family in low socioeconomic position, house ownership, and substandard living resources based on data from the 2005 Census. Numbers do not always have the same total because of the missing value. CHEER, Children’s Health and Environmental Research; GM, geometric mean; SEP, socioeconomic position.

**Figure 1:**
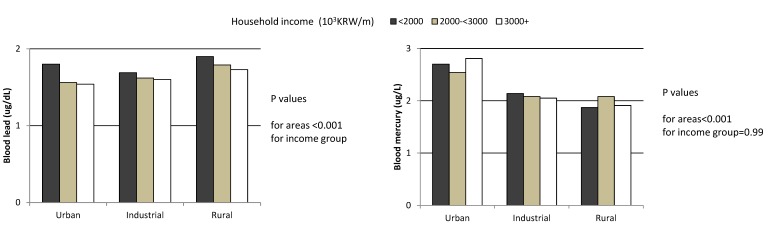
Levels of blood lead and mercury by household income at enrollment and residential areas in the 2281 children, CHEER, Korea, 2005–2006. Geometric mean levels for blood metal were calculated using the multivariate generalized linear model after adjusting for age, gender, child’s weight, second hand smoke, survey year, hour for playing outside (for blood lead), and fish consumption frequency per week (for blood mercury).

**Table 3:** Association between high levels of blood lead or mercury and the individual socioeconomic position adjusted for or stratified by the deprivation index of the community in 2281 children at enrollment, CHEER, 2005–2006, Korea.

Heavy Metals	Risk of High Level of Heavy Metal	Adjusted for DI				Stratified by DI						
						Less Deprived Community			More Deprived Community			*p*-Interaction
		N	OR	95% CI	*p*-Trend ^†^	N	OR	95% CI	N	OR	95% CI	
		(≥Median/<Median)				(≥Median/<Median)			(≥Median/<Median)			
Lead	Household income (×103KRW/month) ^‡^											
	<2000	436/341	1.54	1.22–1.95	0.0003	245/170	1.20	0.88–1.63	191/171	1.88	1.26–2.79	0.4745
	2000–2999	409/415	1.23	0.99–1.54		272/242	1.04	0.79–1.38	137/173	1.52	1.02–2.26	
	≥3000	299/381	1.00	Ref.		223/240	1.00	Ref.	76/141	1.00	Ref.	
	Educational attainment of father (years)											
	<12	69/35	2.18	1.36–3.50	0.0112	37/22	1.40	0.76–2.59	32/13	2.88	1.27–6.53	0.1873
	12	499/474	1.10	0.91–1.34		328/270	0.96	0.75–1.23	171/204	1.14	0.82–1.59	
	>12	492/576	1.00	Ref.		321/328	1.00	Ref.	171/248	1.00	Ref.	
Mercury	Household income(×103KRW/month)											
	<2000	385/392	0.94	0.74–1.20	0.6347	201/214	1.00	0.73–1.37	184/178	1.14	0.76–1.72	0.2341
	2000–2999	401/423	0.95	0.76–1.20		236/278	0.91	0.68–1.21	165/145	1.26	0.84–1.91	
	≥3000	355/325	1.00	Ref.		241/222	1.00	Ref.	114/103	1.00	Ref.	
	Educational attainment of father (years)											
	<12	41/63	0.64	0.40–1.03	0.0304	24/35	0.79	0.42–1.48	17/28	0.85	0.38–1.91	0.8538
	12	462/511	0.85	0.70–1.04		291/307	1.01	0.79–1.31	171/204	0.88	0.63–1.24	
	>12	587/481	1.00	Ref.		333/316	1.00	Ref.	254/165	1.00	Ref.	

ORs and 95% CIs estimated for the group with median or higher of each heavy metal referenced by the group with less than median of each blood heavy metal level using logistic regression model adjusted for age, gender, child’s weight, residential area, second hand smoke, survey year, hour for playing outside (for blood lead), frequency of fish consumption per week (for blood mercury), and deprivation index (DI) of the community (in adjusted model); DI of the community was calculated from household overcrowding, male unemployment rate, head of family in low socioeconomic position, house ownership, and substandard living resources based on data from the 2005 Census. Less deprived; DI ≤ 1.00, more deprived; DI > 1.00; ^†^ Calculated using the ordinal scale of each individual socioeconomic position variable in the corresponding model. P for interaction obtained from the p value of the interaction term of individual and community level of SEP variables in the corresponding full model; ^‡^ 1 USD equals 1132 KRW as of 04 January 2010. Numbers do not always have the same total because of the missing value. CHEER, Children’s Health and Environmental Research; OR, odds ratio; CI, confidence interval.

**Figure 2:**
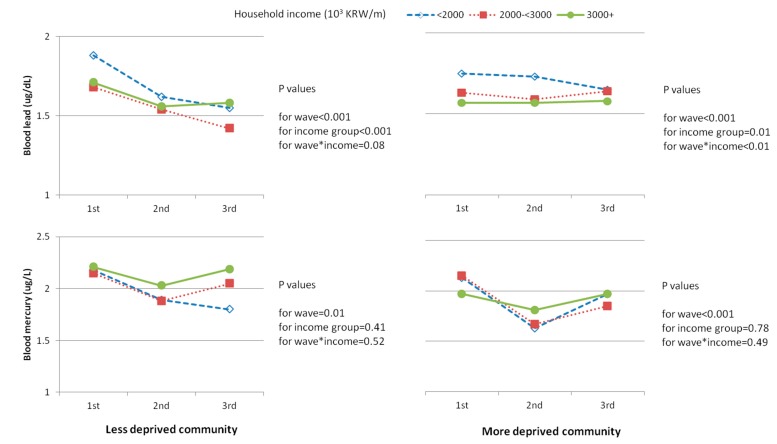
Follow-up trends of blood lead and mercury level by individual and community SEP in 2281 children, CHEER, Korea, 2005–2010; The 1st , 2nd and 3rd waves performed for years of 2005–2006, 2007–2008, and 2009–2010, respectively; Household income; lowest group <2000, middle group 2000–2999, highest group ≥3000 × 10^3^ KRW per month; Deprivation index (DI) of the community was calculated from household overcrowding, male unemployment rate, head of family in low socioeconomic position, house ownership, and substandard living resources based on data from the 2005 Census. Less deprived community: DI ≤ 1.00. More deprived community: DI > 1.00. Geometric mean levels for blood metal were calculated using the mixed model after adjusting for age, gender, child’s weight, residential area, second hand smoke, survey year, hour for playing outside (for blood lead), fish consumption frequency per week (for blood mercury), and baseline blood metal level.

The mean blood mercury did not significantly differ between males and females. However, it was significantly higher in the first survey year and in children living in urban areas, and showed significant increasing trends according to the increase of the father’s educational level in male, and household income in female children. The mercury level of female children was significantly higher in the more deprived communities than the less deprived communities. The level of mercury was higher in those who consumed fish frequently, in a significant dose-response manner (Table S2).

The blood levels of lead and mercury by residential area and household income at enrollment, after adjusting for confounders and covariates, are illustrated in Figure 1. The blood lead levels by residential area significantly differed and were the highest in children living in rural areas, followed by industrial and urban areas. It was also significantly higher in the lower household income groups. Conversely, the mercury level was significantly higher in children from urban areas, while there was no significant difference between different household income groups.

The risks of having high blood levels of lead and mercury are presented in Table 3. The risk of high blood lead levels was significantly higher in the lower individual SEP, with a significant dose-response observed after adjusting for the DI. The association was much stronger (or mainly due to) in the more deprived communities than in the less deprived communities. However, the risk of high blood mercury levels was significantly lower in the lower father’s educational attainment group after adjusting for DI, in a significant dose-response manner, and there were no significant differences between the less and more deprived communities. When the analysis was performed with a generalized linear model using a log transformed continuous scale of blood metal level, similar patterns of associations were obtained (Table S3).

To assess the follow-up trends of the blood levels of lead and mercury, the levels according to the survey waves were adjusted for the covariates, as illustrated in Figure 2. The blood lead level tended to decrease significantly during the follow-up from the first to third wave in the less deprived communities, while it did not show a decreasing trend in the more deprived communities. On the other hand, the blood mercury level tended to decrease significantly during the follow-up both in the less and more deprived areas.

## 4. Discussion

In this study, we found that the risk of high blood lead levels was significantly higher for the lower individual SEP, with a significant dose-response observed, and that the association was much stronger in the more deprived communities. A significantly decreasing tendency for blood lead level as children got older was observed in the less deprived communities, while the trend did not decrease in the more deprived communities. Mercury levels, attributed mainly to the frequency of fish intake, were significantly lower in the lower individual SEP and showed a significant decreasing tendency as children got older; however, there was no significantly different pattern between the less and more deprived communities.

The higher blood lead levels in children in a lower individual or community SEP observed in the present study are consistent with the findings of previous studies [27,28,29]. Individual SEP modulates the neurotoxic effects of lead [30]. In a longitudinal study measuring both prenatal and postnatal blood lead levels and investigating the neurodevelopmental effects of lead, the mean mental developmental index showed an apparent decrease with increasing blood lead levels in the lower SEP group. Furthermore, the detrimental effects of lead on neurodevelopment persisted into young adulthood [31]. Moreover, another study showed that delayed cognitive development with less recovery occurs more frequently in children exposed to higher prenatal and postnatal levels of lead in the less optimal SEP [32]. Nutritional deficiencies of vitamin D, vitamin C, and iron, which may easily occur in a socioeconomically disadvantaged environment, can influence exposure, absorption, and distribution of lead [33].

The increased risk of high blood lead levels in association with lower individual SEP (*i.e.*, a lack of affordability of cleanliness and nutritionally sufficient food, poor housing conditions, and lack of opportunities to obtain information or education related to environmental health for the children’s parents) could be potentiated by lower community SEP through both direct and indirect pathways. As a direct pathway, environmental exposure may increase by residing in deprived areas, which commonly feature heavy traffic related to industrial activities, smelters, waste disposal, and highways or large intersections. As an indirect pathway, community deprivation increases individual vulnerability to environmental exposure by decreasing adaptation capacity to the environmental exposure condition. For instance, in communities with lower SEP, there may not be sufficient resources and services for residents, such as informational and educational deliveries and facilities, which in turn can result in different levels of parents’ and children’s recognition and behavior in relation to environmental health compared to those living in less deprived communities. Avoiding outside play during smoggy days, hand-washing immediately after coming inside, and ventilation after cooking, among others, are examples of environmental health practices that can be effectively applied to reduce environmental exposure by an appropriate delivery of information and education. On the other hand, in more deprived communities with a lack of health care facilities, children have less opportunities to recover from frequently occurring mild health problems and have decreased physiological capacity to remove toxicants from their body [8].

As evidence for indirect exposure pathways in the present study, the blood lead levels were significantly higher in children spending more time playing outside, with a dose-response relationship observed, and children in rural areas played outside more than those in urban areas (percentages of children playing outside ≥1 h a day, 29.9% and 38.1%, respectively). However, the higher blood lead level in rural areas (*versus* urban and industrial areas) in the present study differs from the findings reported in other studies. The rural areas in this study were mostly suburban and socioeconomically deprived areas: the mean DI values were 0.84 in urban areas and 1.71 in rural areas.

Another important finding of this study is that the decrease of the lead level as children got older, from the first to the third wave, was limited to children in the less deprived communities. The decreasing trend of blood lead levels as children age has been previously reported in cross-sectional human biomonitoring [34,35] and longitudinal studies, with an increase from birth to two years followed by a decrease at 10 years [11] and a further 11% decrease from 15 to 17 years [36]. In Korea, the decrease of the blood lead level as a child gets older may be due to a dilution effect of the body burden of exposed lead, as well as an actual decrease of lead exposure in the environment as a result of the Environmental Health Act, which came into effect in 2009 [37]. The lack of decreasing trend in the more deprived communities suggests the possible existence of persistently increased sources of exposure to lead.

The blood mercury levels of the participants in this study were higher than those reported recently in Korea (GM, 1.77 μg/L in male and 1.69 μg/L in female students) [34] and those reported in other countries (0.2–0.4 μg/L) [38,39,40]. Differences in the mercury levels by age or gender were not significant, which is consistent with the finding from a previous study in Sweden [36]. Although the main health outcomes of mercury result from organic mercury exposure, we measured the total blood mercury level, which well represents organic mercury in large population studies. The relation showed an opposite direction compared to blood lead in terms of the association with individual SEP; the level was higher for higher individual SEP. In previous reports, the relationships between mercury levels and individual SEP have been inconsistent, though the majority showed positive relationships. The mercury levels were significantly elevated in the higher education group in the 1999–2000 NHANES study [41] and in pregnant Spanish women [42]. In addition, the mercury levels in hair were elevated in the higher SEP group in one community in Korea in one previous study [14], and the blood mercury level was significantly higher in the higher SEP group in children throughout Korea in another [17]. However, a study conducted in Finland with male adults found that the mercury levels in hair significantly correlated with lower individual SEP [43], while the urinary mercury level did not show a significant relation with several individual SEP indicators in one community in Korea [44].

Although the preference for fish consumption differs by country, fish consumption [14] has been indicated as the main source of organic mercury exposure. People in the Korean peninsula have reported frequent fish consumption [45]. In the present study, the frequency of fish intake was higher for higher individual SEP; the percentages of children who ingested fish 4 times or more per week were 5.1%, 6.0%, and 7.5% in the low, middle, and high household income groups, respectively. However, the overall frequency was higher in the more deprived communities (6.6%) than in the less deprived communities (5.8%) (Table S3), which might be due to the fact that communities located at coastal areas, where fish is easily available, mostly consist of deprived regions in Korea [45]. Therefore, the blood mercury level seemed to be attributed mostly to fish consumption.

This study has some limitations. First, in terms of the descriptive values of blood lead and mercury levels in children, it needs careful interpretation, because the study participants were not enrolled based on a representative sampling frame. The primary purpose of the CHEER study is to examine the possible associations between environmental exposure and health effects in children based on a longitudinal follow-up design. However, the study includes a large number of children, schools, and areas in the whole nation stratified according to the degree of environmental exposure, *i.e.*, urban, industrial, and rural areas. Second, we did not investigate direct exposure sources in the community, such as distance to factories or industrial complex areas, and distance to roads. In addition, we did not measure the actual deprivation status of the study communities, but rather constructed the DI previously developed for studies on health inequality. Third, in terms of the blood metal measurements, the levels were measured only once, and the possibility of measurement error hence exists. Finally, we did not measure the serum iron level in the participating children, which is an important contributing factor of the blood lead level. However, based on the information obtained from the complete blood cell counts, there was no child diagnosed as iron deficiency anemia. Nevertheless, despite these limitations, our study showed significant relationships between blood lead and mercury levels and individual and community SEP levels in Korean children.

## 5. Conclusions

The blood lead level showed a significant inverse association with individual and community SEPs, with synergism. Conversely, the blood mercury level was significantly and positively associated with higher individual SEP and more deprived communities, which seemed to be mostly attributed to fish intake. From a public health point of view, community level interventions with different approaches for different metals are warranted to protect children from exposure to environmental lead and mercury.

## Supplementary Files

Supplementary File 1
