# Supplementary material for: Disparities in Children’s Blood Lead and Mercury Levels According to Community and Individual Socioeconomic Positions
Source: Int J Environ Res Public Health. 2015 May 29;12(6):6232–48. doi: 10.3390/ijerph120606232 (PMC4483698; doi:10.3390/ijerph120606232)
Supplement: Supplementary File 1 [file ijerph-12-06232-s001.pdf]

Supplementary Information

Disparities in Children's Blood Lead and Mercury Levels According to Community and Individual Socioeconomic Positions

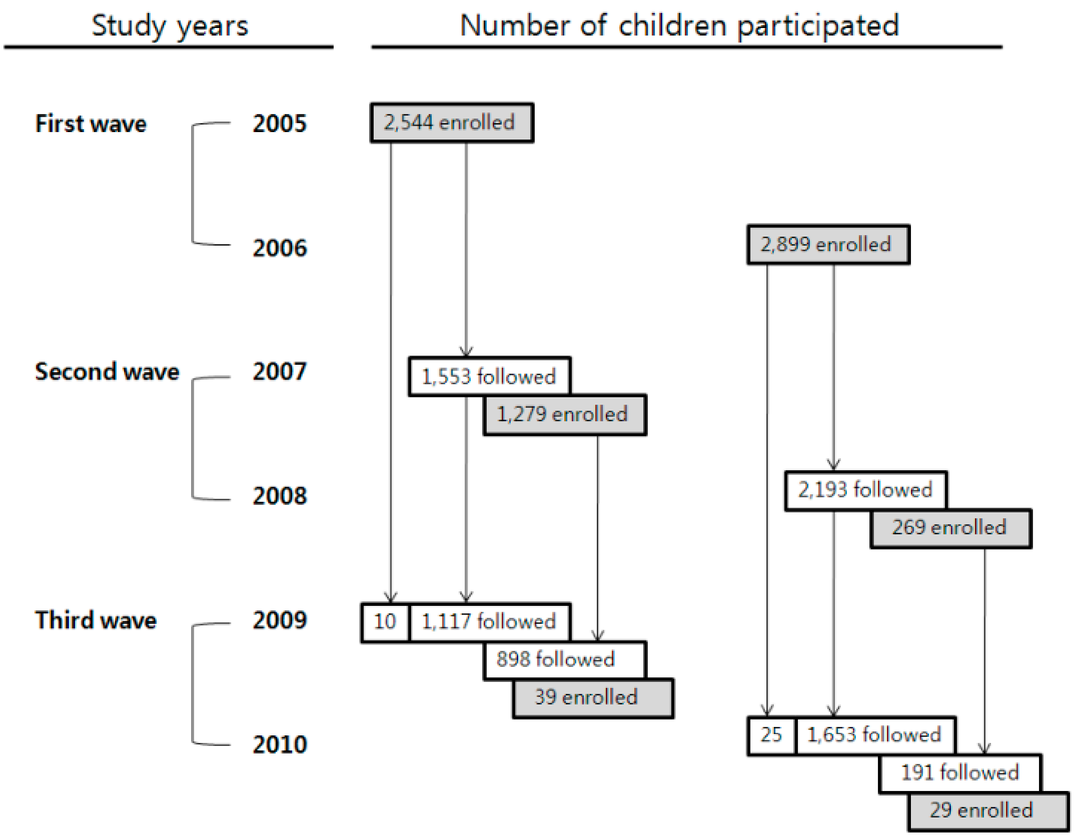

Figure S1. Number of participating primary school children by study years in the CHEER, Korea, 2005–2010.

The number of total enrolled children was 7059. Among them, blood lead and mercury level were measured in 6094 children. During the study period in the three waves, 2770 children were followed 2 times completely. Of 2770, 2285 children have both information of an individual SEP indicator and blood metal level. Finally, 2281 children were selected as a study population after excluding two children who were over eight years old and blood lead or mercury level was outlier.

Table S1. Comparison of children examined at all three waves and the lost to follow-up among children who were 1st or 2nd grades students in elementary schools at baseline and enrolled at 2005 and 2006.

	**Children Followed-up Completely**		**Lost to Follow-up**		***p* Value**
	**No.**	**%**	**No.**	**%**	
All No.	2311	100	1872	100	
Age (years)					
5	0		3	0.2	
6	463	20.0	390	20.8	
7	1464	63.4	1153	61.6	
8	384	16.6	326	17.4	
Mean ± SD	6.97 ± 0.60		6.96 ± 0.62		0.8663
Gender					
Male	1157	50.1	1010	54.0	
Female	1154	49.9	862	46.0	0.0123
Residential area					
Urban	765	33.1	480	25.6	
Industrial	880	38.1	855	45.7	
Rural	666	28.8	537	28.7	<0.0001
Household monthly income					
Lowest	778	34.1	562	31.8	
Middle	825	36.1	599	33.9	
Highest	680	29.8	606	34.3	0.0093
Baseline blood lead (μg/dL) GM ± GSD	1.65 ± 1.76		1.65 ± 1.76		0.8430
Baseline blood mercury (μg/L) GM ± GSD	2.06 ± 2.04		2.03 ± 2.09		0.4839

Table S2. Relations between frequency of fish intake, blood mercury level, and individual and community SEP in 2281 children, CHEER, Korea, 2005–2006.

**Mercury Level (ug/L), GM**		**Fish Intake ***		**(%) of Children, Frequent Fish Intake**
		**Rarely**	**Frequently**	
Less deprived community		(5.70)	(6.40)	(5.8)
	low	1.94	2.00	(4.2)
Household income	middle	1.93	2.12	(5.3)
	high	1.83	2.28	(7.9)
More deprived community		(6.12)	(7.03)	(6.6)
	low	2.07	2.39	(6.0)
Household income	middle	2.05	2.22	(7.0)
	high	2.00	2.42	(6.7)

* Fish intake: rarely < once a week, frequently 4 or more times a week.

Table S3. Generalized linear regression analyses in association between individual and community level of SEP and blood lead and mercury levels in 2281 children, CHEER, Korea, 2005–2006.

□	**Adjusted by DI**				**Stratified by DI**								***p*-Interaction ‡**
					**Less Dedprived**				**More Deprived**				
	**N**	**% Increase**	***p*-Value**	***p*-Trend †**	**N**	**% Increase**	***p*-Value**	***p*-Trend †**	**N**	**% Increase**	***p*-Value**	***p*-Trend †**	
Household income (×10^3 KRW/month) §													
<2000	777	−3.90	0.6343	0.6782	412	7.43	0.1187	0.1271	362	10.45	0.0128	0.0083	0.4684
2000–2999	824	−6.41	0.1804		514	−1.40	0.7374		310	1.47	0.7141		
≥3000	680	reference			463	reference			217	reference			
Educational attainment of father (years)													
<12	104	24.76	0.0004	0.0358	59	16.35	0.1048	0.7443	45	31.62	0.0003	0.0084	0.5174
12	973	0.72	0.7903		598	−2.83	0.4625		375	3.32	0.3427		
>12	1068	reference			649	reference			419	reference			
Household income (×10^3 KRW/month)													
<2000	777	4.85	0.6490	0.6508	412	−1.14	0.8305	0.8395	362	0.80	0.9431	0.4198	0.2609
2000–2999	824	2.66	0.6695		514	0.97	0.8430		310	−2.64	0.5795		
≥3000	680	reference			463	reference			217	reference			
Educational attainment of father (years)													
<12	104	−14.28	0.2220	0.1754	59	−12.62	0.2016	0.0931	45	7.43	0.1187	0.7263	0.8247
12	973	−7.37	0.1944		598	−5.72	0.1752		375	−1.40	0.7374		
>12	1068	reference			649	reference			419	reference			

Parameter estimated for the log transformed heavy metal using multivariate generalized linear regression model adjusted for age, gender, child's weight, residential area, second hand smoke, survey year, hour for playing outside (for blood lead), frequency of fish consumption per week (for blood mercury), and deprivation index (DI) of the community (in adjusted model); DI of the community was calculated from household overcrowding, male unemployment rate, head of family in low socioeconomic position, house ownership, and substandard living resources based on data from the 2005 Census. Less deprived; DI ≤ 1.00, more deprived; DI > 1.00; † Calculated using the ordinal scale of each individual socioeconomic position variable in the corresponding model; ‡ 1 USD equals 1132 KRW as of 04 January 2010. Numbers do not always have the same total because of the missing value. CHEER, Children's Health and Environmental Research; OR, odds ratio; CI, confidence interval.

© 2015 by the authors; licensee MDPI, Basel, Switzerland. This article is an open access article distributed under the terms and conditions of the Creative Commons Attribution license (http://creativecommons.org/licenses/by/4.0/).
